# Ultrasound-Guided Mandibular Nerve Block in the Infratemporal Fossa for Awake Craniotomy: A Cadaveric Feasibility Study

**DOI:** 10.7759/cureus.112047

**Published:** 2026-07-04

**Authors:** Miguel Mayol Del Valle, Natasha Frontera, Adriana Vazquez-Medina, Eric J Perez-Perez, Giovanny E Perez, Esteban R Rivera, Alejandro E Cedeño-Morán, Francisco Del Valle, Diego Troche, Gloria Carrasquillo-Rosa, Ashlie Maldonado-Pérez, Nivia Perez, Elird Bojaxhi

**Affiliations:** 1 Neurosurgery, University of Puerto Rico, Medical Sciences Campus, San Juan, PRI; 2 Surgery, University of Puerto Rico, Medical Sciences Campus, San Juan, PRI; 3 Otolaryngology, Head and Neck Surgery, University of Puerto Rico, Medical Sciences Campus, San Juan, PRI; 4 Medicine, University of Puerto Rico, Medical Sciences Campus, San Juan, PRI; 5 Pediatrics, Hospital Episcopal San Lucas, Ponce, PRI; 6 Anatomy and Neurobiology, University of Puerto Rico, Medical Sciences Campus, San Juan, PRI; 7 Anesthesiology, Mayo Clinic, Jacksonville, USA

**Keywords:** awake craniotomy, cadaveric study, infratemporal fossa, mandibular nerve block, neuroanesthesia

## Abstract

Awake craniotomies are an essential neurosurgical technique, allowing surgical intervention in adjacent areas while preserving eloquent brain areas such as Brodmann areas 1-4, Broca's area, and Wernicke's area. Central to the success of awake craniotomy is the provision of safe and effective analgesia throughout the procedure. The traditional scalp block for an awake craniotomy involves anatomical landmark localization of six nerves: supraorbital, supratrochlear, zygomaticotemporal, auriculotemporal, and greater and lesser occipital nerves. Employing adjunctive techniques via trigeminal nerve branch blocks can maximize nerve-impulse blockade in areas of interest during this procedure. To study a novel and potentially viable technique of local anesthesia administration to the mandibular branch of the trigeminal nerve (V3), we studied four adult cadaveric specimens preserved in 70% alcohol. Two out of four cadaveric specimens underwent bilateral infratemporal fossa (ITF) dissection to confirm the innervation of the temporal muscle and visualize the regional anatomy. The remaining two cadaveric specimens underwent dye injection (5 mL of methylene blue) within the target areas of the ITF. Injections were guided by a linear ultrasound probe, placed parallel to the zygomatic arch. Two fossae were injected in the interfascial plane of the superior and inferior heads of the lateral pterygoid muscle, while the other two fossae received injections between the periosteum of the infratemporal fossa and the fascia of the lateral pterygoid muscles. The correct injection site was confirmed by direct dye visualization after dissection of the infratemporal fossa. Our cadaveric study demonstrates a potential local distribution of anesthesia when the ultrasound-guided injection is placed below the zygoma, deep in the plane between the periosteum of the infratemporal fossa and the fascia of the lateral pterygoid muscles. Dissection of the ITF following dye injection demonstrated dye distribution at the level of the entrance of V3 at the foramen ovale. The use of ultrasound guidance allows for the administration of methylene blue dye within the ITF, reaching deep territories, including the V3 entrance. This novel technique offers a promising approach to improve scalp block for awake craniotomies by enabling anesthesia of V3 using ultrasound guidance. However, additional clinical studies are required to evaluate the efficacy and safety of this approach.

## Introduction

In neurosurgery, the awake craniotomy is an essential technique that balances surgical intervention with the preservation of eloquent brain functions, such as Brodmann areas 1-4, Broca’s area, and Wernicke's area [1]. A retrospective study by Groshev and colleagues revealed that patients undergoing awake craniotomies experienced fewer neurological deficits (7%) compared to those undergoing craniotomy under general anesthesia (23%) [2]. The advent of this technique, now often employed in cases of brain tumors in close proximity to eloquent areas or for localizing seizure foci, represented a paradigm shift in neurosurgical practice [1]. Additionally, functional brain mapping during awake craniotomy has been increasingly performed at several institutions, underscoring the need for the optimization of this procedure [3]. However, an adequate pain-free experience in patients undergoing awake craniotomies has yet to be achieved.

A key factor for ensuring the success of awake craniotomies is achieving safe and effective anesthesia throughout the procedure [4]. Traditionally, local anesthetics and sedatives have been administered during critical stages of the surgery, such as the application of cranial pins and the removal of the bone flap. Exploring innovative ways to reduce intraoperative pain, allowing for the minimization of sedative and narcotic use during awake neurosurgical procedures, is essential. The traditional scalp block for an awake craniotomy involves localizing six anatomical landmarks: supraorbital, supratrochlear, zygomaticotemporal, auriculotemporal, greater, and lesser occipital nerves [5]. However, blockage of the auriculotemporal nerve alone, made with anatomical landmark guidance, is insufficient to provide surgical anesthesia to the deep tissues of the temporalis region during an awake craniotomy [6]. Employing adjunctive techniques via trigeminal nerve branch blocks can optimize nerve impulse blockade in targeted areas during an open craniotomy. One alternative is the mandibular nerve (V3) block, which gives rise to dural branches, the auriculotemporal branch, and the posterior and anterior deep temporal branches. This study proposes an improvement to the traditional scalp block by targeting V3 using ultrasonography rather than the traditional auriculotemporal nerve block guided by anatomical landmarks for improved anesthesia in awake craniotomies, particularly addressing the anesthesia of deep temporal tissues. Employment of ultrasonography enables real-time nerve and vessel imaging, precise needle placement, and effective injectate distribution [7]. Employment of ultrasound and anatomical landmarks to inject methylene blue into the infratemporal fossa (ITF) of cadavers may achieve adequate distribution of local anesthesia to V3. This technique may help reduce pain for patients undergoing temporalis muscle dissection during awake craniotomies, therefore reducing the need for sedation and intraoperative narcotic use.

## Technical report

This cadaveric study was exempt from IRB approval. Local and international ethical guidelines and laws that pertain to the use of human cadaveric donors in anatomical research were followed and respected during this study. In order to demonstrate the feasibility of this technique to the existing scalp block that focuses on blocking V3, two primary objectives were established: (1) to demonstrate anatomical reliability and (2) to use the anatomy to introduce an adjunctive technique for V3 nerve block for use in awake craniotomies. All cadaveric dissections and dye injections were performed by the same operator or under their direct supervision in order to maintain standardization.

Cadaveric dissections

**Figure 1:**
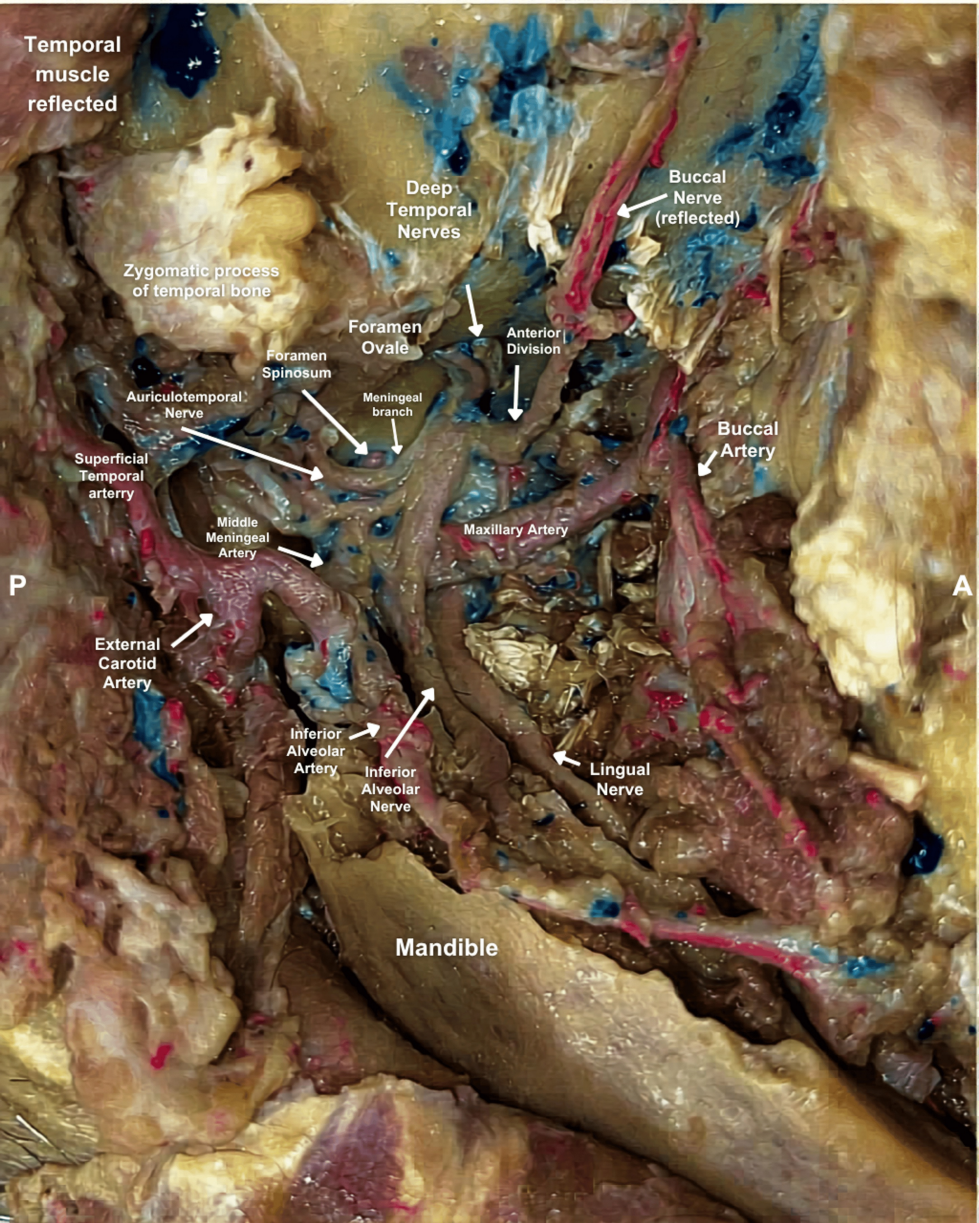
Right infratemporal fossa dissection performed for anatomical reference and planning. A: anterior, P: posterior, V3: Mandibular branch of trigeminal nerve (mandibular nerve).

**Figure 2:**
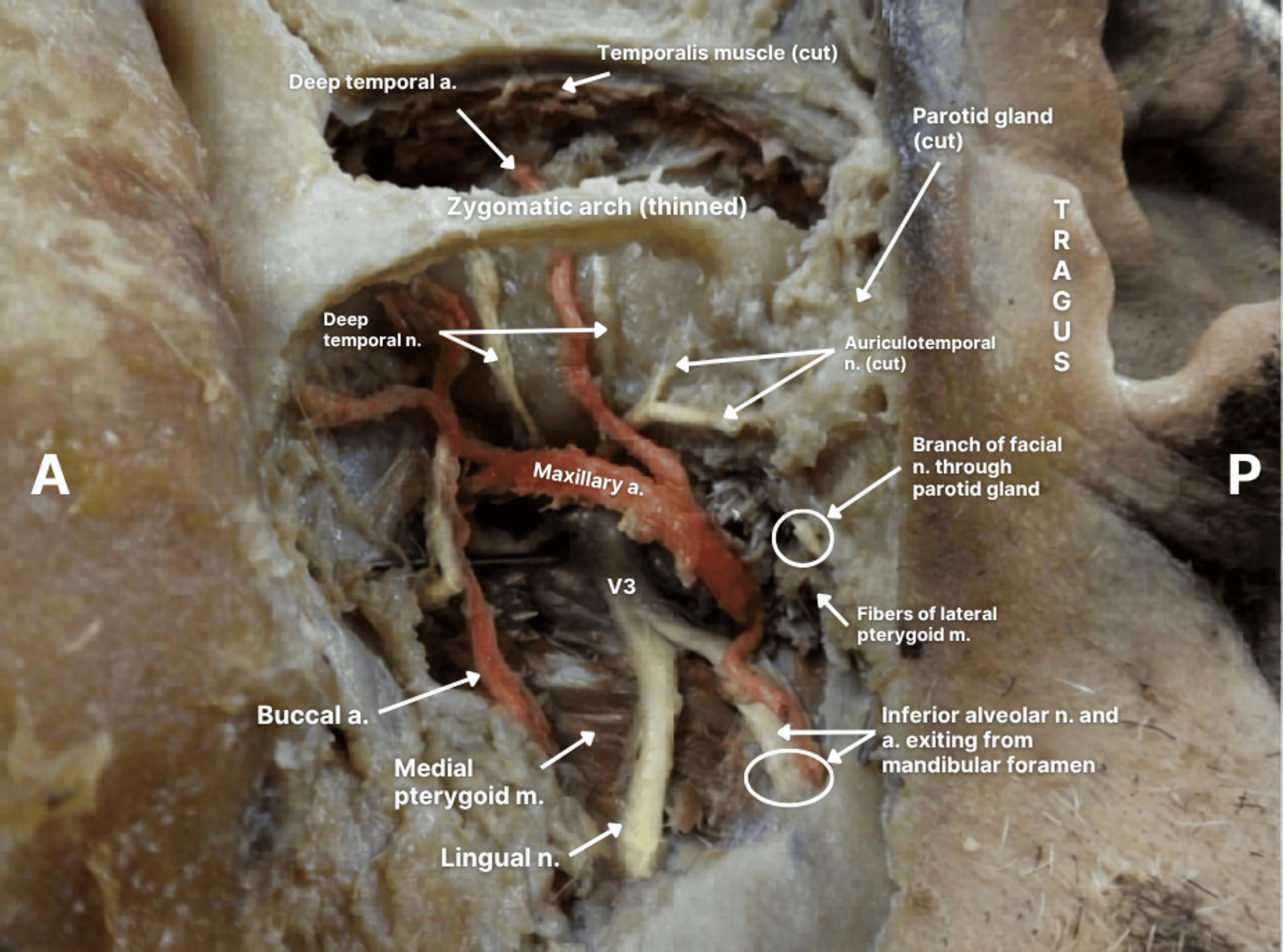
Left infratemporal fossa dissection performed for anatomical reference and planning. a.: artery, n.: nerve, m.: muscle.

Four cadaveric heads preserved in 70% alcohol were provided by the Anatomical Board of Puerto Rico. A total of eight ITFs, two per cadaveric head, were intervened in this study. In the initial stage, four ITFs underwent dissection for comprehensive visualization and anatomical description (Figures 1, 2). In the second stage of our study, we used the knowledge gained from the initial anatomical dissections to perform an experimental methylene blue injection into our targets of interest and later dissected them to assess the dye's distribution.

Soft tissue dissection

Dissection of the ITF was achieved via an extracranial approach. All specimens were rigidly fixed in a Mayfield Head Clamp (Integra LifeSciences Corporation, Cincinnati, OH, USA) and positioned at a 45° angle on the axial plane to the contralateral side of the ITF being dissected. A skin incision was made from the inferior edge of the angle of the mandible, extending superiorly and anteriorly to the auricle into the temporal region, finishing at the hairline. The skin flap was reflected anteriorly, with special attention to the superficial structures, including the temporalis fascia, parotid gland, and the temporal, zygomatic, and buccal branches of the facial nerve. The maxillary artery and its branches were found nested between the medial and lateral pterygoid muscles (Figure 2).

Osseous structure removal

Bony structures, including the zygomatic arch, were thinned with a high-speed drill to enhance the visualization of the ITF’s structures. The superior aspect of the mandible (coronoid process and condyle) was removed using a surgical saw, preserving the lower two-thirds up to the angle’s level. In one of the dissections, we drilled a minimal portion of the inferior border of the zygomatic arch to the level of the floor of the middle cranial fossa (Figure 2). The upper portion of the zygomatic arch was preserved in order to provide a clearer understanding of the relationship between this bony landmark and the underlying neurovascular structures.

Deep tissue dissection

The lateral pterygoid muscle and the inferior portion of the temporalis muscle were dissected out. Special care was taken to preserve the main branches of the maxillary artery and the V3 of the trigeminal nerve. Through this dissection, the sensory innervation of the temporalis muscle was reconfirmed by visualizing multiple branches of V3 penetrating the muscle (Figure 2). We also noted that the anterior and posterior deep temporal nerves originate at the level of the foramen ovale as well as the auriculotemporal nerve (Figure 1). Careful dissection between the pterygoid musculature was performed using microscissors and forceps to identify the regional vessels, specifically the maxillary artery. This served as a guide to identify feeders to the temporalis muscle at the level of the ITF. The inferior alveolar nerve was identified as it exited from the mandibular foramen, at the medial surface of the mandible, and followed superiorly up to the skull base (Figure 2), merging with the main trunk of V3 at the level of the foramen ovale (Figure 1). Deep tissue dissection allowed identification of other notable branches, such as the auriculotemporal nerve and the roots innervating the temporalis muscle (Figures 1, 2).

Radiographic planning

**Figure 3:**
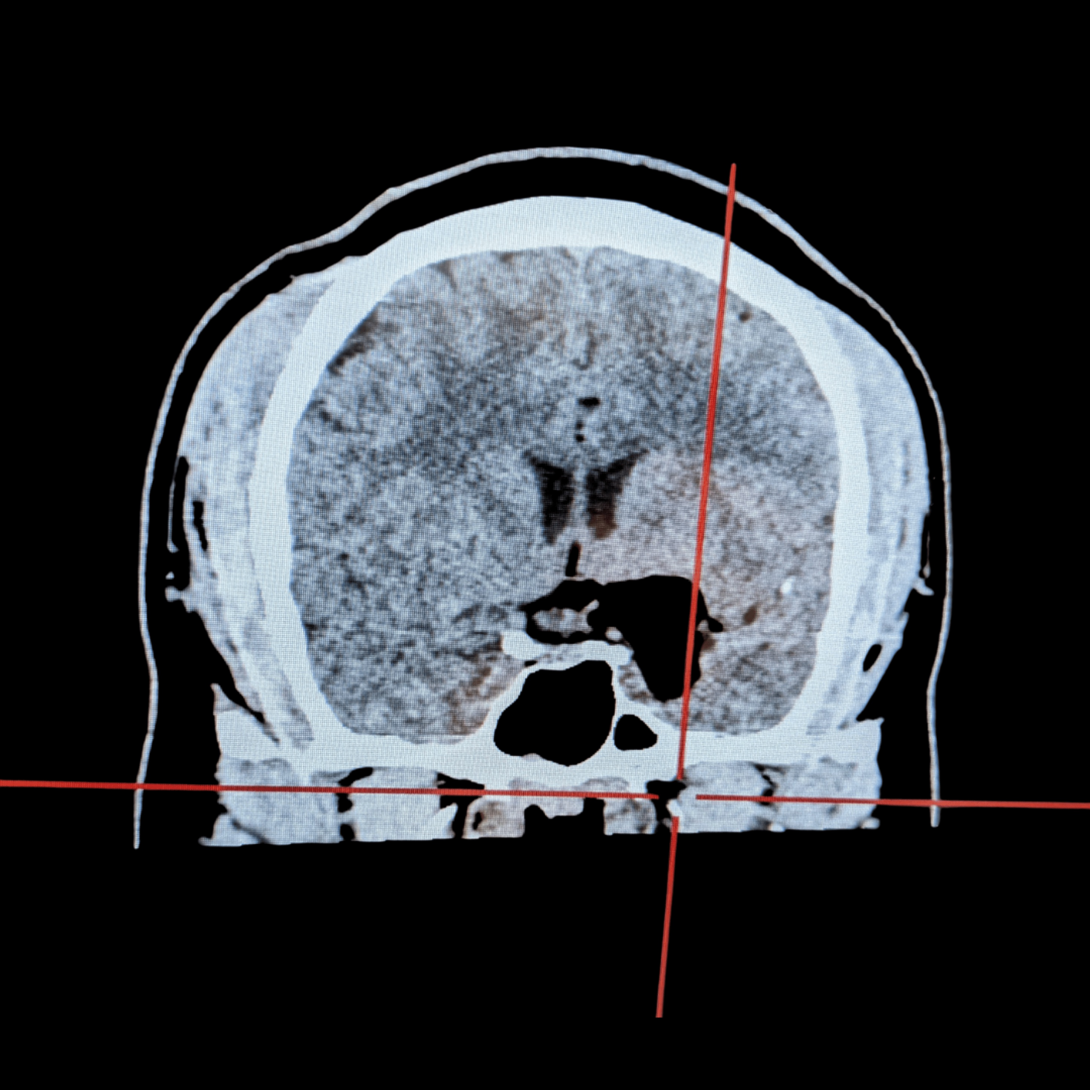
CT scan of the cadaver’s head, coronal view. Red cross is marking the foramen ovale and probable path of V3.

We performed head computed tomography (CT) on our cadaveric heads and utilized a Medtronic StealthStation™ Surgical Navigation (Medtronic Navigation, Inc., Louisville, CO, USA) to study the anatomy radiologically. This correlation allowed us to better visualize the anatomy and to assist in choosing our injection trajectory, as would be performed during surgical planning. We identified the exit point of the trigeminal nerve from the cranium through the foramen ovale (Figure 3).

Injection procedure

**Figure 4:**
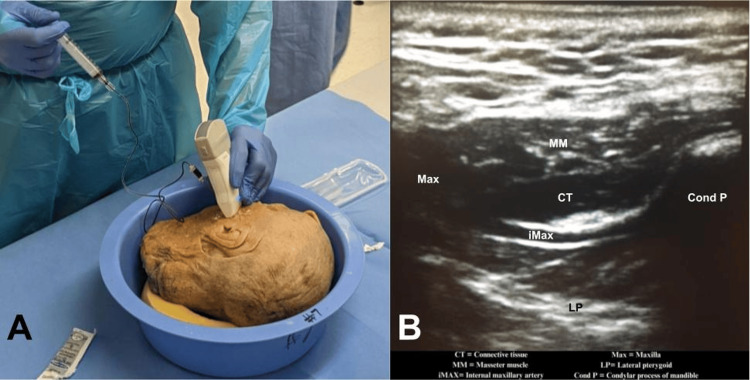
Ultrasound-guided methylene blue injection and cadaver ultrasound image showing anatomical landmarks. A) Representation of ultrasound-guided methylene blue injection. B) Ultrasound image of the cadaver head with probe placed parallel to the zygomatic arch showing anatomical landmarks. CT: connective tissue, Max: maxilla, MM: masseter muscle, iMax: maxillary artery, LP: lateral pterygoid, Cond P: condylar process of mandible.

We utilized a concentrated aqueous solution of methylene blue (Sigma-Aldrich, St. Louis, MO, USA) to outline the potential distribution of anesthesia in the fascial plane of the V3. Two cadaveric specimens underwent four percutaneous injections of methylene blue, one into each ITF. A linear ultrasound probe was placed parallel to the zygomatic arch, and a 25-gauge needle was placed perpendicular to the skin (Figure 4A), 3 cm anterior to the tragus. After confirming correct needle positioning using anatomical landmarks, we introduced the needle approximately 3.0-3.5 cm into the ITF. The needle trajectory was monitored with the ultrasound probe throughout the entire process (Figure 4B). Two distinct targets were tested. One tested target was the space between the fascial planes of the superior and inferior heads of the lateral pterygoid muscles. The second target was the space between the periosteum of the ITF and the fascia of the lateral pterygoid muscle. Each site was tested in two cadaveric ITFs by injecting a single 5 mL dose of methylene blue into each location, utilizing ultrasound guidance for targeting.

Confirmation of correct injection of methylene blue

**Figure 5:**
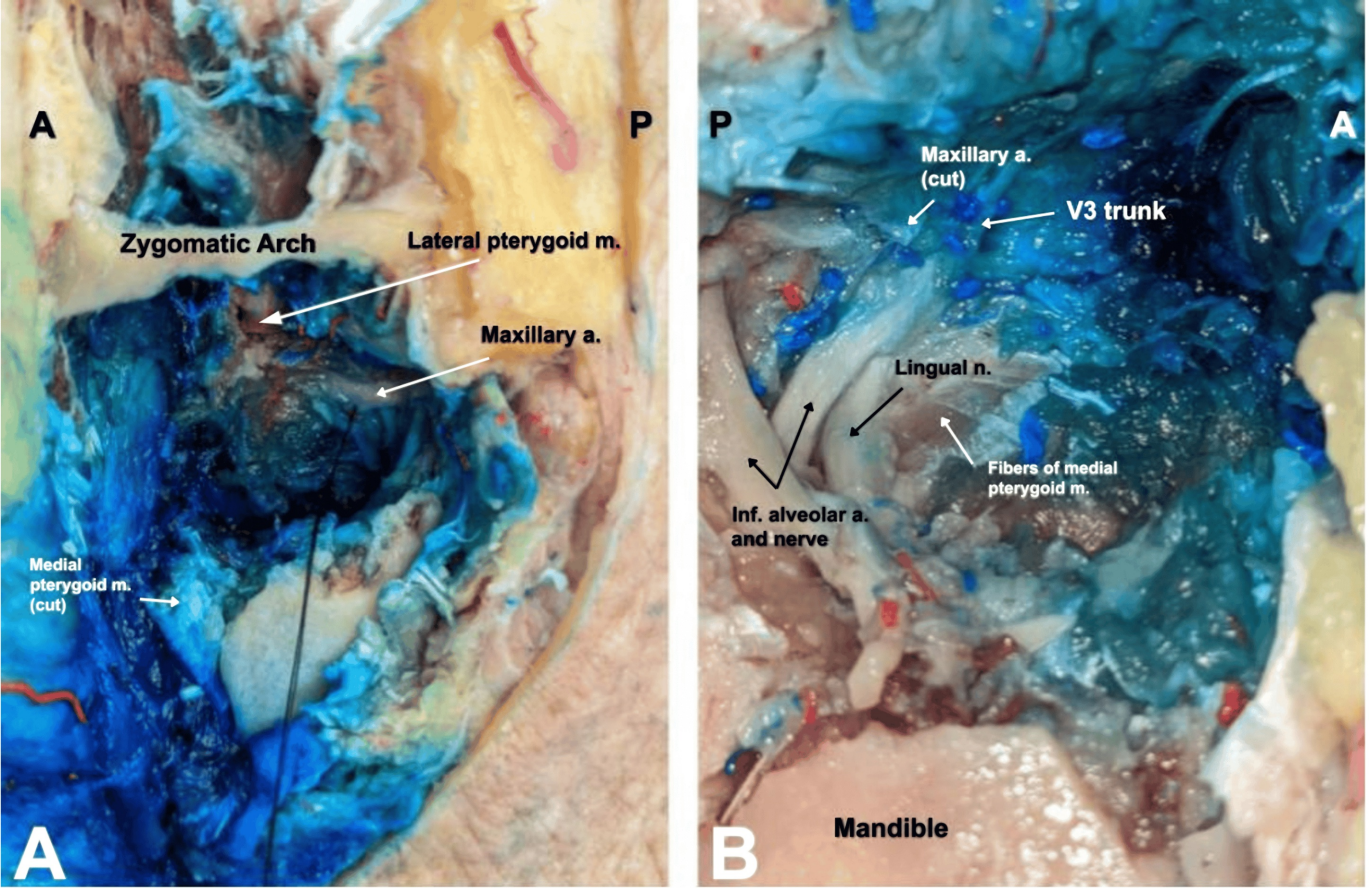
Methylene blue spread following ultrasound-guided injections in the lateral pterygoid and infratemporal planes. A) Spread of methylene blue after injection in the interfascial plane of the superior and inferior heads of the lateral pterygoid muscles. B) Spread of methylene blue after injection between the periosteum of the infratemporal fossa and the fascia of the lateral pterygoid muscles. a.: artery, m.: muscle, n.: nerve, Inf.: inferior, A: anterior, P: posterior.

After the dye was injected, we immediately dissected the ITF as described above. Correct dye placement was confirmed by the visualization of a blue discoloration throughout the fossa (Figure 5).

Results

In our study, we performed eight cadaveric dissections. Four dissections were not injected with methylene blue and were compared with those described in the literature; the dissections confirmed the expected anatomy of the ITF. Our dissections yielded results similar to those reported by Pouwels and colleagues [8]. They performed a cadaveric study and described the anatomic findings of the deep temporal nerve of 10 hemifaces [8]. They found that the number of deep temporal branches varied from 2 (30%) to 3 (70%) per side. The mean distance to the tragus varied from 40 to 53 mm, and the mean distance from the cranial portion of the posterior root of the zygomatic bone to the deep temporal nerves varied from 29 to 35 mm.

The anterior division of V3 is subdivided into nerves that specifically innervate the buccal, masseteric, temporalis, lateral pterygoid, and medial pterygoid muscles, which are crucial for functions such as facial expression, chewing, and jaw movement [9]. In our cadaveric dissection, we observed deep temporalis muscle nerve branches emerging from the anterior division (Figure 1). The posterior division of V3 splits into two branches, the inferior alveolar nerve and the lingual nerve (Figure 1). The lingual nerve provides sensory innervation to the anterior two-thirds of the tongue, allowing for taste perception and speech. The inferior alveolar nerve enters through the mandibular foramen, situated in the internal aspect of the mandible (Figure 2). Before entering the foramen, it gives rise to nerves that innervate the mylohyoid and anterior belly of the digastric muscle, contributing to movements of the hyoid bone and floor of the mouth. Once inside the foramen, it innervates the teeth, while the mental nerve exits the mental foramen to provide sensation to the front mandibular teeth. Before the posterior trunk of V3 splits, it gives off the deep temporal nerves, the meningeal branch, and the auriculotemporal nerve. This last has five main branches: the anterior auricular, articular, parotid, superficial temporal, and branches to the external auditory meatus. These nerves collectively provide sensation to the temporomandibular joint, the ear, and the scalp, and play a role in secretory and vasomotor innervation of the parotid gland [10]. During dissection of the fossa, the maxillary and superficial temporal arteries were observed branching from the external carotid artery (Figure 1).

**Figure 6:**
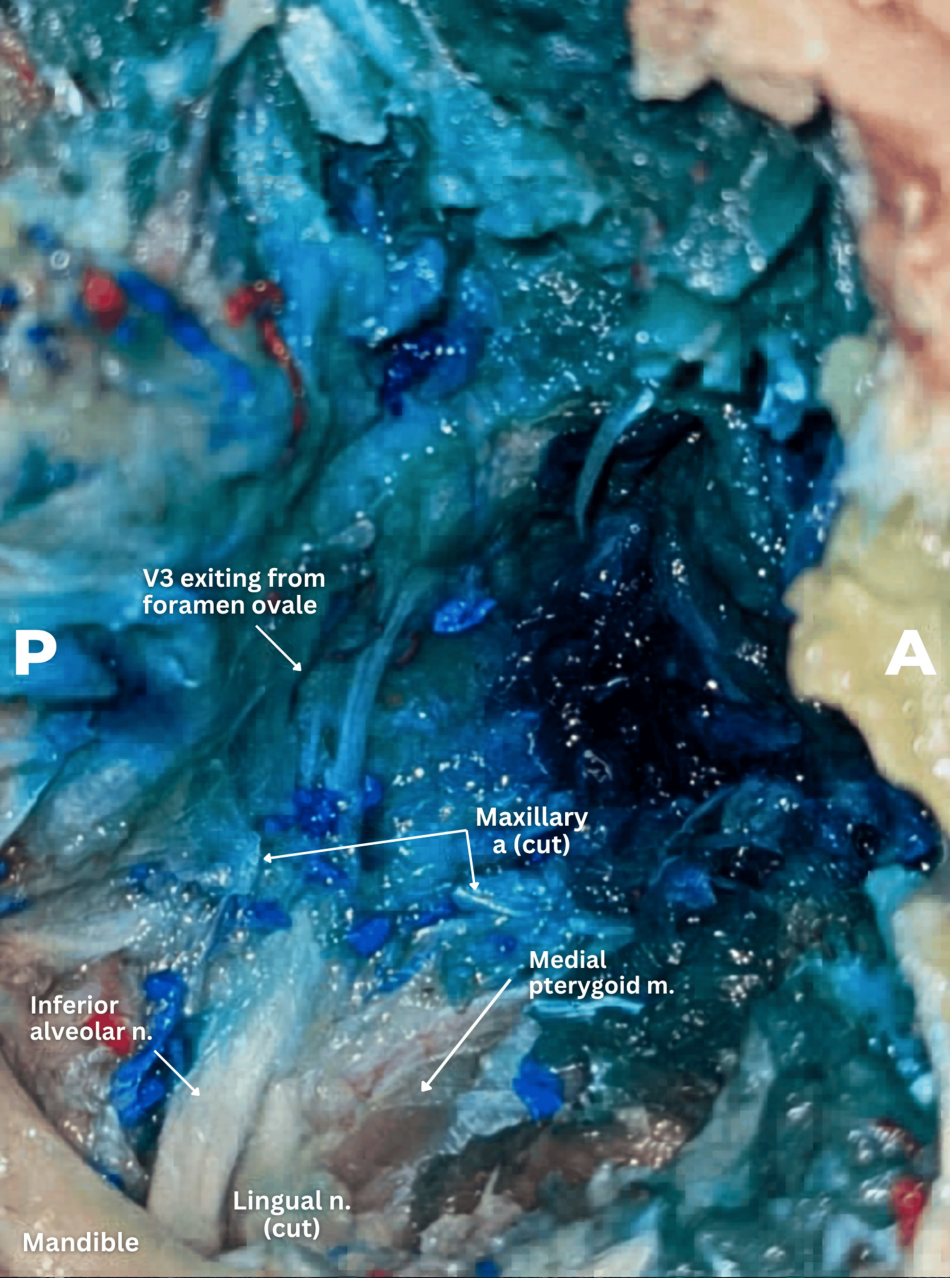
Right Infratemporal fossa dissection after injection between the periosteum and the fascia of the superior head of the lateral pterygoid muscle. V3: Mandibular nerve, a.: artery, m.: muscle, n.: nerve, A: anterior, P: posterior.

We visualized successful dye distribution in two of the four ITFs injected with methylene blue. We performed the injections anterior to the tragus of the external ear and either (a) in the interfascial plane of the lateral pterygoid muscles (Figure 4A) or (b) deep in the plane between the periosteum of the ITF and the fascia of the superior belly of the lateral pterygoid muscles (Figure 4B). Visualization of the injected dye was possible after the removal of the pterygoid muscles. The dissection of the specimen receiving the injection of methylene blue in the interfascial plane of the pterygoid muscles demonstrated that the dye was distributed in the fossa. However, the dye did not appear to reach the foramen ovale, the auriculotemporal nerve, or the deep temporal nerves. A complete dissection of deeper structures was not performed after visualizing a lateral pterygoid muscle that lacked methylene blue staining, suggesting that the dye did not penetrate deeper structures (Figure 4A). In the specimen that received the injection in the plane between the periosteum and the fascia of the lateral pterygoid muscles, the dye is seen distributed in the fossa, reaching the foramen ovale, and completely covering V3 and its branches (i.e., meningeal branch, deep temporal nerves, auriculotemporal nerve, masseteric nerve, lingual nerve, buccal nerve, lateral and medial pterygoid nerves, and the inferior alveolar nerve) (Figures 5B, 6).

## Discussion

This study explores a technique for supplemental V3 blockade during scalp blocks for use in awake craniotomies. Adequate analgesia remains a challenge during awake craniotomies, and ultrasound-guided injection of V3 within the ITF may prove to be a safe procedure and enhance patients’ comfort during surgery. The feasibility of this injection site was evaluated by injecting methylene blue in the infratemporal fossa of two cadaveric specimens, followed by in-depth cadaveric dissections of the ITF. These dissections revealed three key facts that suggest the application of the lateral infrazygomatic V3 block may be a beneficial adjuvant to the traditional scalp block: 1. The maxillary artery lies lower than the middle cranial fossa (i.e., V3 exit point) and permits a safe corridor for ultrasound-guided lateral approach to the V3 block, decreasing the risk of vascular injury [1]. 2. The sensory innervation of the temporalis muscle and scalp in the temporal region originates at the level of the foramen ovale, through V3. 3. It was thought that the origin of V3 was in a fascial plane between the medial and lateral pterygoid muscles. However, our dissections demonstrate the origin is deep to the pterygoid musculature, between its fascial plane and the periosteum of the ITF. Thus, a proper sensory block may be achieved with an anesthetic injection deep to the lateral pterygoid muscle.

The use of a V3 nerve block in postoperative pain management for patients undergoing surgery for oropharyngeal carcinoma has shown promise in reducing pain and opioid use [11]. Furthermore, studies suggest that V3 anesthesia may provide superior patient comfort compared with conventional auriculotemporal anesthesia techniques, particularly during temporomandibular joint (TMJ) arthrocentesis [12]. Potential complications associated with this technique include inadvertent injury to important structures, such as the maxillary and middle meningeal vessels and the facial nerve, which may lead to hemorrhage, hematoma formation, facial nerve palsy, oculomotor palsy, masseter muscle weakness, and auditory tube vertigo [13]. Providing adequate anesthesia to the deep temporal nerves and meningeal branches of V3 can reduce the need for intraoperative muscular and dural injections. This, in turn, will reduce intraoperative pain and decrease the risk of inadvertently injecting anesthesia into the brain, which could provoke seizures. The approach proposed in this study aims to mitigate these risks while ensuring adequate analgesia, thereby reducing the need for additional analgesic intervention in subsequent stages. This application may improve the current standard of care for regional anesthesia in awake craniotomies, as an adjunct or replacement for the traditional scalp block. Clinical studies are needed to confirm that injecting anesthetic agents into the space between the periosteum of the ITF and the fascia of the lateral pterygoid muscles reaches all branches of V3 and offers superior analgesia during scalp blocks.

The significance of achieving adequate anesthesia perioperatively cannot be overstated, as it directly correlates with intraoperative comfort, pain management, and postoperative recovery. Chaki and colleagues reported that 19% of patients experienced incisional pain upon emergence from anesthesia, which escalated further toward the end of the conscious procedure [4]. This underscores the limitation of the traditional scalp block for intraoperative pain management. Traditional scalp block procedures may not provide sufficient pain control because they do not effectively anesthetize target areas, particularly the temporalis muscle and dura. While the temporal region is supplied primarily by the zygomaticotemporal nerve anteriorly and the auriculotemporal nerve posteriorly, small, infiltrating branches originating from V3 are often overlooked. Bojaxhi et al. demonstrated that the temporalis muscle proved highly sensitive to dissection away from the periosteum during surgical exposure [14]. Targeting only the auriculotemporal and zygomaticotemporal nerves may have resulted in insufficient blockade of the underlying deep tissues. Chaki et al. reported a reduction in subjective pain, opioid use, and somnolence with ultrasound-guided nerve block for local anesthesia when compared to general anesthesia in patients undergoing craniotomies [4]. Some authors advocate maximizing the use of local anesthesia techniques (scalp blocks) with ultrasound-guided injections as adjuncts to perioperative protocols to optimize postoperative recovery after craniotomy [4,14]. Our study offers insights into an entry point below the zygoma, situated between the periosteum of the ITF and the fascia of the pterygoid musculature. Targeting V3 nerve during scalp blocks for awake craniotomies may optimize analgesia and enhance patient comfort.

A study by Sato and Nishiwaki investigated the accuracy of a landmark method with traditional scalp blocks and found that failure of zygomaticotemporal nerve blockade was due to difficulty reaching its deep distribution [15]. In our study, injecting the dye below the zygoma, between the periosteum of the ITF and the fascia of the lateral pterygoid muscles, proved to reach deeper structures, such as the temporal branches of V3. This suggests that sufficient anesthesia to the temporal muscle should be provided when using the proposed technique. However, further studies are needed to evaluate new approaches to the maxillary nerve block.

This study enabled direct visualization of ITF anatomy across multiple cadaveric models and the planning of an injection technique, which may serve as a route for anesthetic delivery into the ITF for use in V3 blockage to improve analgesia during awake craniotomies. However, this study is not without its limitations. One such limitation we cannot assume adequate analgesia, a subjective experience, based solely on the visualized distribution of a colored dye. Differences in dye flow between cadavers and live patients must be considered. It is understood that since cadavers lack a functioning cardiorespiratory system and have lower temperatures, the density and resistance of tissues are higher than in live humans. Which, in turn, may affect the flow of dye through embalmed tissue. Despite this, the difference in tissue density observed is less pronounced than expected. Studies employing elastography, a modality used to evaluate tissue stiffness, have demonstrated that the flow of embalming fluid along tissue planes follows the path of least resistance, similar to live patients [16]. Additionally, our study is limited by a small sample size of four cadaveric specimens with eight ITFs, of which four were used for anatomical exploration and four underwent dye injection into the respective anatomical targets. For this reason, additional studies are needed to test our proposed technique, including injections into cadaver specimens fixed with other materials and fresh specimens more closely resembling live patients. Finally, an inherent limitation of cadaveric studies, particularly of subjective experiences such as analgesia, is that they are limited in the clinical conclusions that may be drawn on the benefit of proposed interventions. Therefore, following further cadaveric validation, the proposed technique would require further in vivo validation, potentially in a multicenter clinical trial to evaluate its safety and efficacy.

## Conclusions

The traditional auriculotemporal nerve block could be improved by developing complementary techniques to minimize intraoperative pain and postoperative opioid use. Targeting V3 within the ITF utilizing ultrasound guidance may improve analgesia of the cutaneous tissue and deeper muscular soft tissue, such as the temporalis muscle. Our cadaveric study demonstrates a potential local distribution of anesthesia when the ultrasound-guided injection is placed below the zygoma, deep in the space between the periosteum of the ITF and the fascia of the lateral pterygoid muscles that may adequately reach deep tissue, including V3 at the foramen ovale. If proper anesthesia can be achieved with this technique, it may lead to improved analgesia during awake craniotomies, shorter recovery times, and shorter hospital stays. However, additional studies are required to evaluate the efficacy and safety of this unique approach in fresh cadaveric specimens or specimens fixed with other materials.
